# Polarized Water Driven Dynamic PN Junction-Based Direct-Current Generator

**DOI:** 10.34133/2021/7505638

**Published:** 2021-01-24

**Authors:** Yanghua Lu, Yanfei Yan, Xutao Yu, Xu Zhou, Sirui Feng, Chi Xu, Haonan Zheng, Zunshan Yang, Linjun Li, Kaihui Liu, Shisheng Lin

**Affiliations:** ^1^College of Microelectronics, College of Information Science and Electronic Engineering, Zhejiang University, Hangzhou 310027, China; ^2^State Key Lab for Mesoscopic Physics and Frontiers Science Center for Nano-optoelectronics, Collaborative Innovation Center of Quantum Matter, School of Physics, Peking University, Beijing 100871, China; ^3^Guangdong Provincial Key Laboratory of Quantum Engineering and Quantum Materials, School of Physics and Telecommunication Engineering, South China Normal University, Guangzhou 510006, China; ^4^State Key Laboratory of Modern Optical Instrumentation, Zhejiang University, Hangzhou 310027, China

## Abstract

There is a rising prospective in harvesting energy from the environment, as in situ energy is required for the distributed sensors in the interconnected information society, among which the water flow energy is the most potential candidate as a clean and abundant mechanical source. However, for microscale and unordered movement of water, achieving a sustainable direct-current generating device with high output to drive the load element is still challenging, which requires for further exploration. Herein, we propose a dynamic PN water junction generator with moving water sandwiched between two semiconductors, which outputs a sustainable direct-current voltage of 0.3 V and a current of 0.64 *μ*A. The mechanism can be attributed to the dynamic polarization process of water as moving dielectric medium in the dynamic PN water junction, under the Fermi level difference of two semiconductors. We further demonstrate an encapsulated portable power-generating device with simple structure and continuous direct-current voltage output of 0.11 V, which exhibits its promising potential application in the field of wearable devices and the IoTs.

## 1. Introduction

Sustainable energy harvesting from the environment is always required to meet the increasing energy demand of modern information society [1–5], especially the burgeoning Internet of Things (IoTs) and bioelectronic devices [6–21]. As a clean and abundant mechanical source, water flow energy is the most potential candidate in our daily life. For macroscale and ordered movement of water, it has been used to generate electricity since the development of Faraday generator, which is widely used in hydropower industry until now [22, 23]. However, for microscale and unordered movement of water, collecting energy from flowing water molecules and converting it into electricity is still challenging, which can be used as in situ energy or sensor in some special environments. Recently, water has been used to generate electricity by placing water moleculars over carbon nanotube [24–27], graphene [28–32], polymer [33–36], or other nanostructured materials [37–39]. Various physical mechanisms have been proposed to explain the water energy harvesting phenomenon based on the flow of water, such as the moving liquid dragging electrons [24, 30], water evaporation [38, 39], liquid flow induced triboelectrification [40–42], and charges fluctuating asymmetric potential in substrate [25, 43]. However, those generators cannot continuously output direct current especially under the low frequency and unordered water movement, which is limited by the moving direction. And the device output is still not high enough to drive the load element, requiring for high performance direct-current generators with some novel physical mechanism.

The semiconductor physics are well developed, and thus the interaction between water and semiconductor could provide an insight into the electricity generation by moving water droplets on or inside various materials. Actually, water is mysterious as it remains a quantum material where quantum fluctuation has a great impact on its appearance [44]. The highly polarized water molecules can work as conductive dielectric medium, where the distribution of electron density resembles the picture of black holes [45]. Recent studies about the dynamic metal-semiconductor junction [46–48], semiconductor-semiconductor junction [49–51], and other semiconductor system [52–61] provide an inspiration to explore the electronic dynamics at the dynamic water/semiconductor junction interface [62, 63]. In particular, we have proposed the physical phenomenon of the carriers rebounding in the dynamic junction generator with ultrahigh built-in electric field at the semiconductor interface [49–51], which can instantaneously polarize the water molecules in the semiconductor interface. Herein, we design a dynamic PN junction generator with water as a moving dielectric medium between two semiconductors, which has been rarely investigated before [64, 65].

In this generator, the water droplet is sliding between a P-type silicon and a N-type silicon with different Fermi levels, which leads to a continuous current output. The physical mechanism is proposed to be originated from the dynamic polarization process of moving water molecules between semiconductors, in which polarized water work as a moving dielectric medium between the dynamic PN junction. In detail, when water molecules contact with silicon, the sandwiched water molecules are instantaneously polarized, and the free charge carriers in silicon are accumulated at the water-semiconductor interface, and then they reach the electrostatic balance and polarization balance, which is driven by the Fermi level difference between two semiconductors. As water droplet moves, the polarization balance is repeatedly broken and reestablished. Meanwhile, these induced polarized electrons and holes are released and rebound to the P-type and N-type semiconductors, respectively. In this way, such PN water junction with the dynamic polarized water-semiconductor interface generates continuous voltage or current in the external circuit. The direct-current generator based on the dynamic water-semiconductor junction with polarized water as moving dielectric medium realizes open-circuit voltage of up to 0.3 V and short-circuit current of 0.64 *μ*A, with matched internal resistance with the traditional electronic information devices based on the PN junction. We further demonstrated an encapsulated portable power generation device with simple structure and sustainable direct-current electricity. Our approach reveals the quantum polarization properties of water molecular in the dynamic junction and provides a novel and promising way of converting low-frequency unordered mechanical energy into sustainable direct-current electricity, especially the abundant water droplet flow energy around the world, which is a potential candidate for integrated and miniaturized generator.

## 2. Results and Discussion

**Figure 1:**
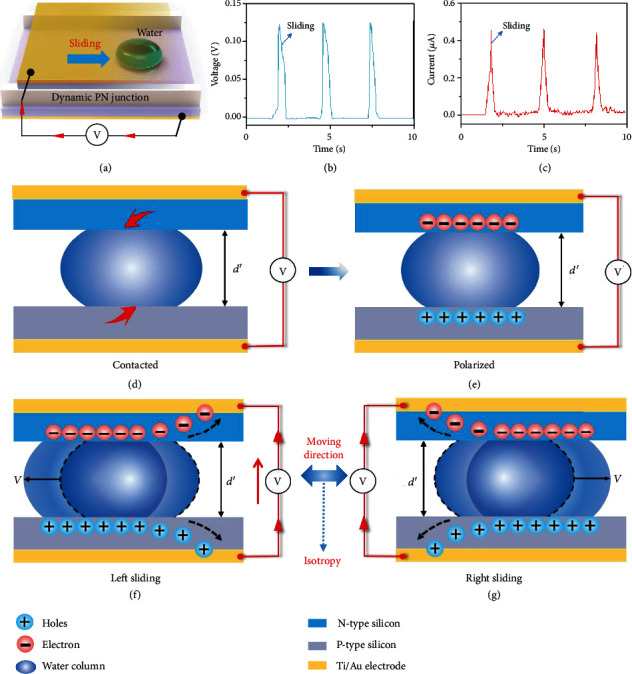
The structure schematic and carrier kinetics of the dynamic PN water junction generator. (a) The three-dimensional diagram of the dynamic liquid-semiconductor junction structure generator, which consists of two semiconductors, Ti/Au electrodes, liquid, and PVC layer. The curves of the (b) voltage generation and (c) current generation depends on time. Each peak of voltage and current stands for an independent process of the water droplet movement. Schematic drawing showing the working mechanism and charge transport process of the dynamic PN water junction generator under (d) contacted, (e) polarized, and (f) left and (g) right sliding states. The hole and electron are represented in positive and negative sign, respectively. With the movement of water droplet, these interfacial holes and electrons are released and generate directional current output, which shows the characteristics of isotropy in the moving direction and electricity.

The three-dimensional diagram of the dynamic semiconductor-water-semiconductor structure generator is shown in Figure 1(a), in which the water droplet is located between a N-type silicon and a P-type silicon. Two polyvinyl chloride (PVC) layers with a thickness of 1 mm separate two semiconductors, which forms a small channel for water droplet. Liquid can move in this channel in a forward or backword direction. The generated current is collected by the Ti/Au electrodes deposited on unpolished silicon surfaces in the external circuit. Those liquids whose molecular structure is asymmetric can be used in the dynamic water-semiconductor junction generator. Here, deionized water is preferred for its extreme abound and easy availability in our daily life, such as rivers, lakes, oceans, and even our bodies. In the dynamic PN water junction generator, every time a water droplet flows through the channel, a current or voltage signal is generated. As shown in Figure 1(b) and (c), three peaks of voltage with maximum of 0.12 V (current with maximum of 0.46 *μ*A) results from the independent motion process of water droplet with volume of 50 *μ*L at speed of 150 mm/s.

The origin of this electricity generation is attributed to the dynamic polarized water in the junction, which leads to the rebound or reflecting effect of the induced polarized charges. In this semiconductor-water-semiconductor structure, when water droplet contacts with two silicon wafers as shown in Figure 1(d), the water molecules are polarized instantaneously at the water-semiconductor interface driven by the Fermi level difference between two silicon wafers, as shown in Figure 1(e). As water droplet moves along the semiconductors, the dynamically polarized electrons and holes at the interface are rebound to two semiconductors, respectively. In this electricity generation process, the current output particularly shows the characteristics of symmetrical output, without being restricted by the moving direction, as shown in Figure 1(f) and (g). The unique isotropy of the dynamic silicon-water-silicon generator arises from the intrinsic Fermi level difference of two semiconductors, leading to the directional water-semiconductor polarization, which can be used to harvest those low-frequency unordered water flow mechanical energy.

In this dynamic PN water junction generator, the simulation schematic diagram of the polarization process is demonstrated in Figure S1 according to the physical mechanism above. The water molecules are first placed horizontally between the P-type silicon and N-type silicon. Under the Fermi level difference between two semiconductors, water molecules form an ordered arrangement, whose hydrogen atoms point to the N-type silicon and oxygen atoms point to the P-type silicon. After reaching the dynamic stable state, holes can be induced and accumulated in the interface of P-type silicon, and electrons can be induced and accumulated in the interface of N-type silicon, respectively. With the movement of water droplet, these interfacial carriers are released and generate directional current output. The polarized water works as a moving dielectric medium in this dynamic junction [64, 65], which has a negligible damage to the semiconductor interface.

**Figure 2:**
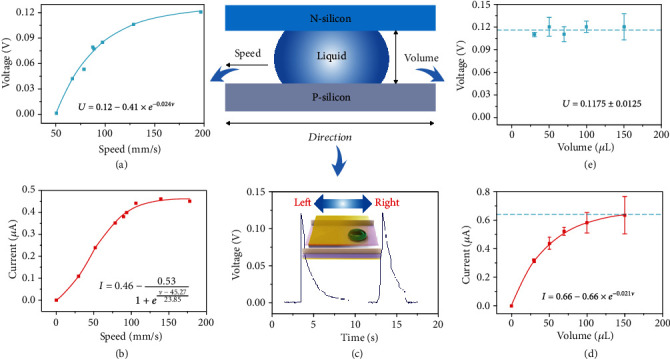
The output of the dynamic water-semiconductor junction generator. (a) The output voltage and (b) current depend on the speed of silicon wafer moving, on the condition of water volume of 50 *μ*L. (c) The curve of the voltage output when the silicon wafer moves to the left or right at a speed of 150 mm/s (water droplet volume is 50 *μ*L). The relationship between the water volume and the (d) output current, (e) output voltage, when the silicon wafer moving speed is 150 mm/s.

According to the aforementioned mechanism, the output characteristic of generator can be determined by the dynamic polarization degree of water molecules, which should be related to these parameters of speed, direction, and volume. As shown in Figure 2(a) and (b), with the increasing speed of the water (water volume of 50 *μ*L), the output voltage and current positively increase and reach saturation values of 0.12 V and 0.46 *μ*A, respectively, at the speed up to 150 mm/s. The moving speed is measured with the system as shown in Figure S2. The voltage and current curves are fitted precisely with a function of *U*_oc_ = 0.12 − 0.41 × e^−0.024*v*^ and *I*_sc_ = 0.46 − 0.53/(1 + e^0.042*v*−1.9^), respectively. The increase in voltage and current output is caused by the changing polarization degree of water at different moving speeds. Whether it is moving to the left or right (the silicon wafer moving speed is 150 mm/s, and the water droplet volume is 50 *μ*L), the generator always generates a positive voltage of 0.12 V from P-type silicon to N-type silicon, as shown in Figure 2(c). Maintaining a constant wafer moving speed of 150 mm/s, the output current increases positively with the water droplet volume and reaches a maximum of 0.64 *μ*A at the volume of 150 *μ*L, as shown in Figure 2(d). The current curve can be fitted precisely with an exponential function of *I*_sc_ = 0.66 − 0.66 × e^−0.021*V*^. The increase in the output current is caused by the polarization of more water molecules per unit time. However, the output voltage nearly remains constant at 0.1175 ± 0.0125 V with the increase of water volume, as shown in Figure 2(e). We have explored the contact angle of the water droplet on the PN junction. As shown in Figure S3, the contact angle of the 50 *μ*L water droplet with silicon is 35.9°, and the voltage output is independent of the contact angle of water and silicon. This constant voltage must be caused by the limitation of the Fermi level difference between two semiconductors, which will be explored later.

These system experiments confirm the output of the dynamic water-semiconductor junction generator is positive related to the degree of dynamic water polarization, which is determined by its speed or volume and overcomes the limitation of the moving direction of liquid, demonstrating the proposed mechanism. To explore the stability of the device against the environmental temperature and humidity, we also have carried out experiments on the relationship between the voltage of the dynamic PN water junction generator with the humidity and temperature of environment. As shown in Figure S4, with the temperature increasing, the voltage output of the dynamic PN water generator decreases, which is caused by enhanced water polarization degree under the higher temperature, but the voltage behaves limited change in the humidity of 22%-54%, which indicates that the humidity is ignorable for outputting electrical signal in our dynamic PN water generator.

**Figure 3:**
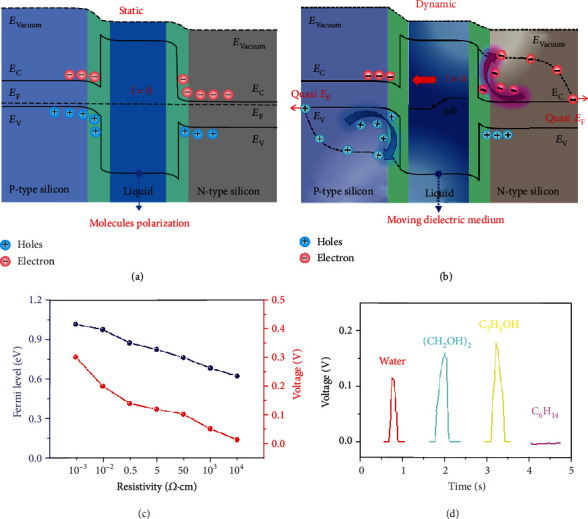
The in-depth exploration about the mechanism based on Fermi level height and molecule polarization. (a) The band structure schematic and carrier kinetics of the static semiconductor-liquid-semiconductor generator during the contact state. (b) The band structure schematic and carrier kinetics of the dynamic semiconductor-liquid-semiconductor generator during the sliding state. (c) The relationship between the output voltage and the Fermi level difference between N-Si substrates (with different resistivity of 0.001, 0.01, 0.5, 5, 50, 1000, and 10000 *Ω*·cm) and P-Si substrates (with constant resistivity of 0.001 *Ω*·cm). (d) The output voltage of the dynamic semiconductor-liquid-semiconductor generator under different polar liquid including water, C_2_H_5_OH, (CH_2_OH)_2_, and nonpolar liquid C_6_H_14_.

The energy band structure diagram of the static and dynamic P-Si/water/N-Si structure is shown in Figure 3(a) and (b). Water molecules at the water/semiconductor interface are polarized as soon as the contact of water and semiconductor, under the Fermi level difference between the water and semiconductors, as shown in Figure 3(a). And the induced polarized charges make the energy band of the water/silicon surface bend. Due to the boron or phosphorus doping, the Fermi level of the P-type and N-type silicon is near to the valence band and conduction band, respectively. Simultaneously, the polarization also exists when the water slides along the silicon. The polarized water works as a moving dielectric medium in the dynamic junction, which can largely protect the dynamic junction from sliding wear of the interface. As shown in the band diagram of the dynamic structure in Figure 3(b), the dynamic polarization electrons and holes in the interface are rebound to N-type and P-type semiconductors, respectively. During the dynamic polarization process, the quasi-Fermi level of the dynamic silicon-water-silicon junction is in a nonequilibrium state, which is different from the equilibrium Fermi level of the static junction. The potential difference and voltage output of the dynamic PN water generator are highly correlated with the quasi-Fermi level difference of the semiconductor electrodes.

So, the performance of dynamic silicon-water-silicon generators with different Fermi level differences has been measured. N-type silicon wafers with different resistivity of 0.001, 0.01, 0.5, 5, 50, 1000, and 10000 *Ω*·cm are used, keeping the resistivity of P-type silicon unchanged at 0.001 *Ω*·cm in this experiment. The Fermi level of the N-type silicon can be calculated with the formula below: [66].

(1) $$\begin{array}{c} E_{F-N}\approx E_{i}+k_{B}T\ln\frac{N_{D}-N_{A}}{n_{i}}, \end{array}$$

(2) $$\begin{array}{c} \sigma =\frac{1}{\rho }\approx qn\mu_{n}, \end{array}$$

where *E*_*i*_ is the middle value of the band gap, *k*_*B*_ is the Boltzmann constant, *T* is the temperature, *n* is the electron concentration, and *p* is the hole concentration. The *n*_*i*_ is the intrinsic carrier concentration of the semiconductor as large as 1.5 × 10^10^ cm^−3^, *μ*_*n*_ is the electron mobility of the N-Si, and *σ* and *ρ* are the conductivity and resistivity of the N-Si we used, respectively. Therefore, the work function of the N-type silicon with the different resistivity of 0.001, 0.01, 0.5, 5, 50, 1000, and 10000 *Ω*·cm is 4.13, 4.17, 4.27, 4.32, 4.38, 4.46, and 4.52 eV, respectively. In the meantime, the P-type silicon with the resistivity of 0.001 *Ω*·cm is used as the substrate, whose work function is 5.14 eV. So, the Fermi level difference of the P-type silicon with different resistivity is as high as 1.01, 0.97, 0.87, 0.82, 0.76, 0.68, and 0.62 eV here. For the N-type silicon with the different resistivity, the output voltage of the dynamic silicon-water-silicon generator is 0.30, 0.20, 0.14, 0.12, 0.10, 0.05, and 0.01 V, respectively, under the moving speed of 150 mm/s and water volume of 50 *μ*L. It is noteworthy that this voltage of devices with different Fermi level differences is positive related to the corresponding Fermi level differences, indicating that the Fermi level difference of semiconductors plays a key role in the voltage output of the dynamic semiconductor-water-semiconductor junction generator, as shown in Figure 3(c). The detailed voltage output of the dynamic silicon-water-silicon generator with different resistivity is shown in Figure S5. After inserting an insulating layer of 10 nm HfO_2_, the output voltage is enhanced to 0.17 V, as shown in Figure S6, indicating the barrier height of the junction interface is the key parameter of the voltage output.

To prove the dynamic polarization of water molecules is the origin of power generation in dynamic silicon-water-silicon structure, different polar and nonpolar liquid are further used to test the performance of the semiconductor-liquid-semiconductor structure. As shown in Figure 3(d), the polar liquid including water, ethylene glycol ((CH_3_OH)_2_), and ethanol (C_2_H_5_OH) can generate voltage output of 0.12, 0.16, and 0.18 V, respectively, with the moving speed of 150 mm/s and liquid volume of 50 *μ*L. However, the nonpolar liquid such as normal hexane (C_6_H_14_) cannot generate any voltage output, verifying that the dynamic polarization process of water is the key for the electricity output of the dynamic semiconductor-water-semiconductor structure. And the polarization process of liquid is related to its dielectric constant. We assume that the *Q* is constant in the dynamic semiconductor-liquid-semiconductor structure, so the voltage can be calculated as below equation:

(3) $$\begin{array}{c} \text{U}=Q/C=Qd/\varepsilon_{0}\varepsilon_{r}S, \end{array}$$

Where *Q* is the quantity of electric charge, *d* is the distance between two semiconductors, *ε*_0_ is the vacuum permittivity, *ε*_*r*_ is the relative permittivity, and *S* is the area of semiconductor. The relative permittivity of water, (CH_3_OH)_2,_ and C_2_H_5_OH is 78.3, 37.7, and 24.3, respectively. As the voltage output is inversely proportional to the relative permittivity of liquid, the dynamic semiconductor-C_2_H_5_OH-semiconductor generator shows the best performance. So, the liquid must be not fully polarized so the power generation voltage is not high as expected, which requires for a system of materials with larger Fermi levels. We also explore the surface wettability of silicon with different liquid. As shown in Figure S7, when the volume of the liquid is 50 *μ*L, the contact angle of water, (CH_3_OH)_2_, C_2_H_5_OH, and C_6_H_14_ on the Si substrate is 35.9°, 27.6°, 11.3°, and 8.8°, respectively, indicating the surface wetting of the liquid also positive related the electric output. We can further do some hydrophilic treatment of the semiconductor substrate. In consideration of the advantages of water droplet, such as abundance and nonpolluting, water is still the most potential in application.

**Figure 4:**
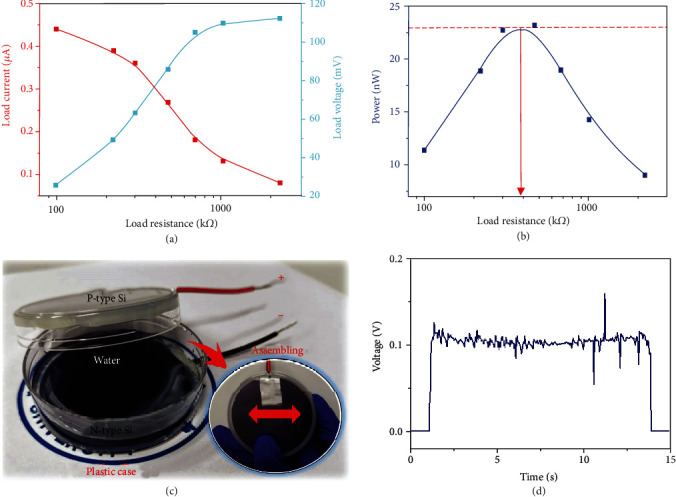
The electricity output and an application demo of the dynamic water-semiconductor junction generator. (a) Voltage and current output of the dynamic silicon-water-silicon generator as a function under a series of different external load resistance. (b) Power output of the dynamic silicon-water-silicon generator as a function with different load resistance. (c) The real device picture of the dynamic silicon-water-silicon generator consists of P-type silicon, N-type silicon wafer, and water. Both the semiconductors are encapsulated in a plastic mold, ensuring the free movement of water between two semiconductors. (d) The direct and continuous voltage output curve of the dynamic silicon-water-silicon generator depends on time.

Compared with the existing water droplet generator, this dynamic PN water junction generator occupies the advantage of sustainable direct-current generating without the limitation of the moving direction. In order to verify the potential application of such generator, the practical work performance of the device under different load resistance has been explored. As shown in Figure 4(a), the output of current and voltage of device varies with the external load resistance. With the increase of load resistance, the current output decreases from 0.44 *μ*A to 0.08 *μ*A, and the voltage output increases from 25.77 mV to 112.12 mV, respectively. Then, the corresponding output of power can be calculated by the product of work voltage and current, as shown in Figure 4(b). According to the equivalent circuit of the device, the maximum power of 22.9 nW has been achieved under the external load resistance of around 390 k*Ω*, indicating the internal resistance of the device is as high as 390 k*Ω*, which is close to the static PN junction device. This low internal-resistance property can largely reduce energy loss, indicating its potential application in the circuit unit energy supply.

Furthermore, in order to reach the practical applications requirement, a simple device demo of the semiconductor-water-semiconductor sandwich structure has been realized. A demo of this dynamic silicon-water-silicon generator is shown in Figure 4(c), which consists of P-type silicon, N-type silicon wafer, and water. Two silicon wafers of 2-inch diameter are glued to the inner surface of a circular plastic mold, and some water is encapsulated between two semiconductors. With the shake of the plastic mold, the water droplets slide freely between two semiconductors and generate continuous direct-current voltage of 0.11 V, as shown in Figure 4(d). This continuity and stability voltage output with limited spikes indicates the potential prospect of the dynamic PN water junction generator in the field of wearable devices and the IoTs. In our further work, the voltage output can be enhanced by designing some microscale array structure or increasing the interfacial barrier height by inserting other dielectric layer in this dynamic PN water generator.

## 3. Conclusion

In this work, we have demonstrated a sustainable direct-current generator base on the dynamic semiconductor-liquid-semiconductor sandwich structure, which can harvest the energy from the mechanical movement of water droplet. The mechanism can be attributed to the dynamic polarization process of water as moving dielectric medium in the dynamic PN water junction, under the Fermi level difference of two semiconductors. During the movement of the water droplet, the polarization balance is broken, and the induced polarized charges are rebounded. Under the effect of the continuously dynamic polarized process, the induced polarized electrons and holes are rebound to N-type and P-type semiconductors, respectively, forming sustainable direct-current electricity. As a representative of the generator, a voltage as high as 0.3 V and current up to 0.64 *μ*A has been achieved based on the silicon-water-silicon structure, with matched internal resistance with the traditional PN junction-based circuit units. As a proof of principle, an encapsulated portable power-generating device with continuous and stable direct-current voltage as high as 0.11 V has been realized, providing a novel and promising way of harvesting widely existed water droplet energy. This dynamic water-semiconductor junction generator reveals the dynamic polarization process in the water-semiconductor interface and shows potential prospect in the field of wearable devices and the IoTs.

## 4. Materials and Methods

### 4.1. Preparation of Silicon Wafers

Single polished N-type silicon wafers with different resistivity are used in the experiment, whose oxide layers are removed by being dipped into 10 wt% HF for 5 minutes. Then water, acetone, and isopropyl alcohol are used to clean up the surface before the electrode fabrication. A layer of Ti with 10 nm and a layer of Au with 50 nm are grown on the unpolished side of the silicon wafer successively with a thermal evaporator, forming a natural ohmic contact electrode with silicon after annealing in 350°C. The same method is applied to fabricate the ohmic contact electrode of single polished P-type silicon wafer.

### 4.2. Physical Measurement Method

The speed of the semiconductor is explored in the electricity generating process. In this experiment, moving water drop is recorded by a high-speed camera from US Phantom called VEO 710 system. Then, the speed is measured with an image processing analysis software called Image-Pro Plus from American MEDIA CYBERNETICS. Keithley 2010 multimeter is used to measure the voltage and current of the generator with the sampling rate of 25 s^−1^, which can be controlled by a LabView-controlled data acquisition system.

## Data Availability

Supplementary materials contain additional data. All data needed to evaluate the conclusions in the paper are present in the paper or the supplementary materials. Reprints and permission information are available online. Readers are welcome to comment on the online version of the paper.

## Supplementary Materials

Supplementary Materials Figure S1. Dynamic polarization process of one water molecule confined between P-type silicon and N-type silicon. (a) The initial simulation structure. Figure S2. Experimental designed system for measuring the moving speed of dynamic PN water junction generator. Figure S3. The contact angle of the water droplet with silicon. Figure S4. The stability of the device against the environmental temperature and humidity. Figure S5. The detail effect of different N-type silicon resistivity on voltage output. Figure S6. Optimization by increasing the interface barrier height. Figure S7. The contact angle of water, (CH_3_OH)_2_, C_2_H_5_OH and C_6_H_14_ on the Si substrate, indicating the surface wettability with different liquid.
