# Supplementary material for: Polarized Water Driven Dynamic PN Junction-Based Direct-Current Generator
Source: Research (Wash D C). 2021 Jan 24;2021:7505638. doi: 10.34133/2021/7505638 (PMC7877395; doi:10.34133/2021/7505638)
Supplement: Supplementary Materials — Figure S1. Dynamic polarization process of one water molecule confined between P-type silicon and N-type silicon. (a) The initial simulation structure. Figure S2. Experimental designed system for measuring the moving speed of dynamic PN water junction generator. Figure S3. The contact angle of the water droplet with silicon. Figure S4. The stability of the device against the environmental temperature and humidity. Figure S5. The detail effect of different N-type silicon resistivity on voltage output. Figure S6. Optimization by increasing the interface barrier height. Figure S7. The contact angle of water, (CH3OH)2, C2H5OH and C6H14 on the Si substrate, indicating the surface wettability with different liquid. [file 7505638.f1.docx]

Supplementary Materials for

Polarized Water Driven Dynamic PN Junction Based Direct-Current Generator

*Yanghua Lu^1^*^†^*, Yanfei Yan^1^*^†^*, Xutao Yu^1^*^†^*, Xu Zhou^2^*^†^*, Sirui Feng^1^, Chi Xu^1^, Haonan Zheng^1^, Zunshan Yang^1^, Linjun Li^3^, Kaihui Liu^2^ and Shisheng Lin^1,3*^*

^1^College of microelectronics, College of Information Science and Electronic Engineering, Zhejiang University, Hangzhou, 310027, P. R. China.

^2^State Key Lab for Mesoscopic Physics and Frontiers Science Center for Nano-optoelectronics, Collaborative Innovation Center of Quantum Matter, School of Physics, Peking University, Beijing, 100871, P. R. China.

^3^State Key Laboratory of Modern Optical Instrumentation, Zhejiang University, Hangzhou, 310027, P. R. China.

^*^Correspondence: [shishenglin@zju.edu.cn](mailto:shishenglin@zju.edu.cn).

† These authors contributed equally to this work

**This file includes:**

Figs. S1 to S7

Figure S1.


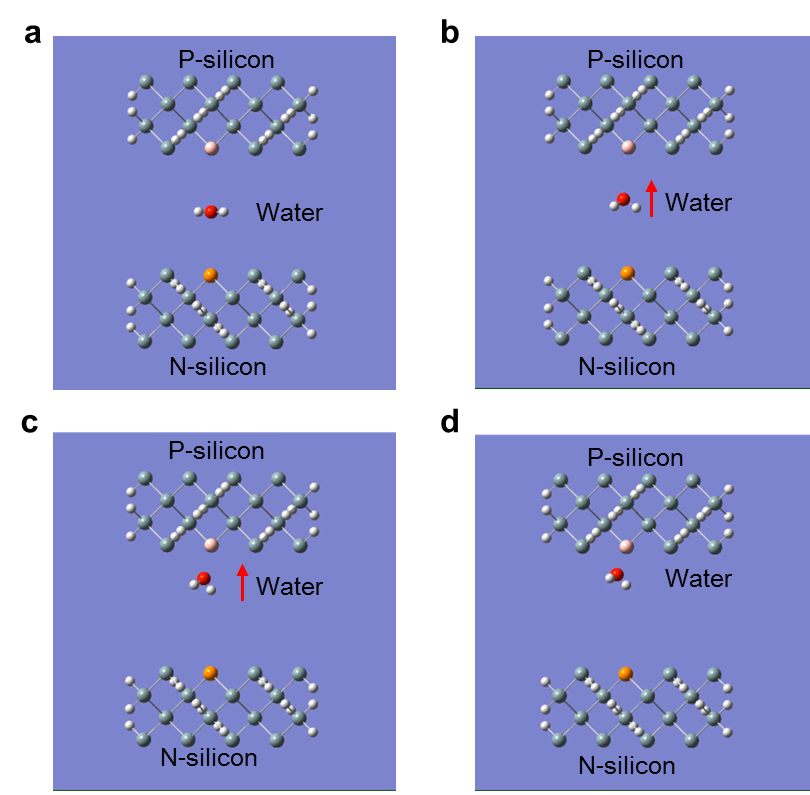


**Figure S1.** Dynamic polarization process of one water molecule confined between P-type silicon and N-type silicon. (a) The initial simulation structure. OH bonds in the water molecule lie horizontally. (b)-(c) Polarization process. (d) Polarized configuration. After the polarization, hydrogen atoms point toward N-type silicon and oxygen atom points toward P-type silicon. White: H; Gray: Si; Red: O; Pink: B; Gold: P.

Figure S2.


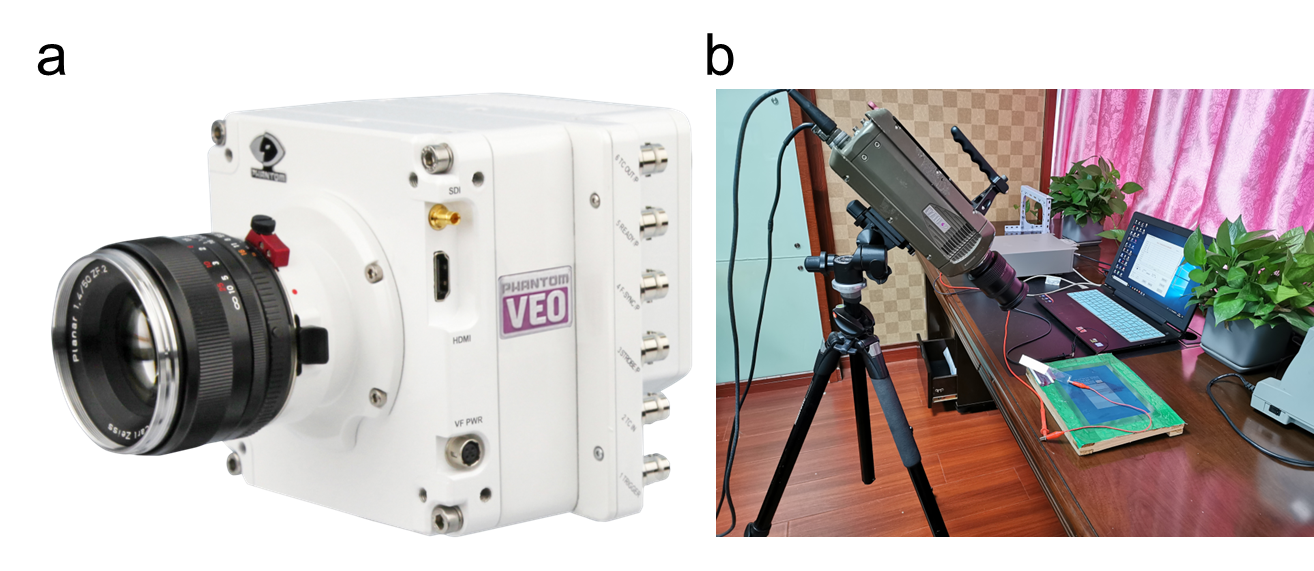


**Figure S2.** Experimental designed system for measuring the moving speed of dynamic PN water junction generator.

Figure S3.


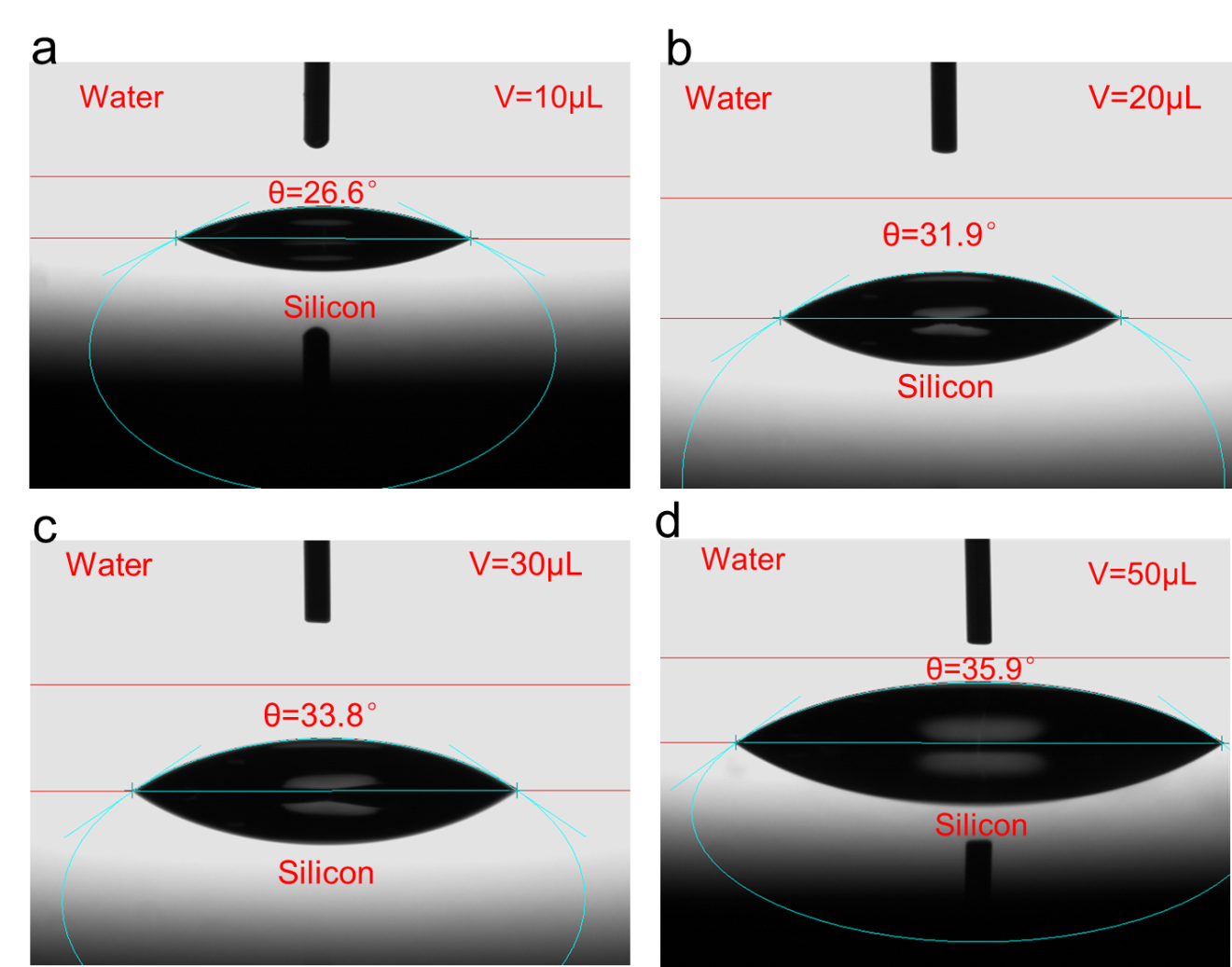


**Figure S3.** The contact angle of the water droplet with silicon.

Figure S4.


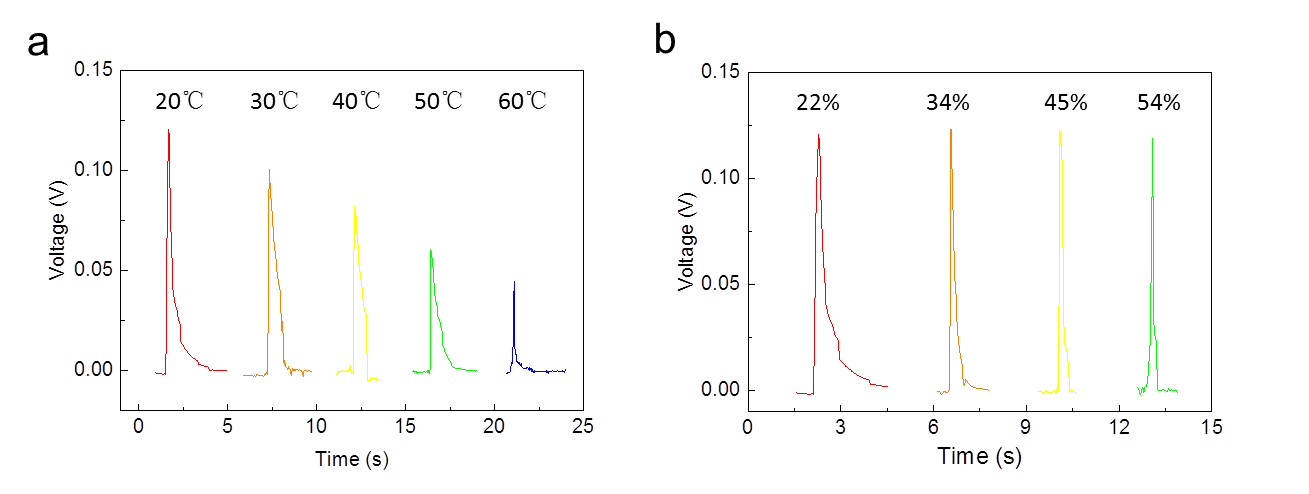


**Figure S4.** The stability of the device against the environmental temperature and humidity. (a) The curve of voltage output when the silicon wafer moves to the left or right at a speed of 150 mm/s (water droplet volume is 50 μL). (b) The direct and continuous voltage output curve of dynamic silicon-water-silicon generator depends on time when the middle water is sliding back and forth continuously.

Figure S5.


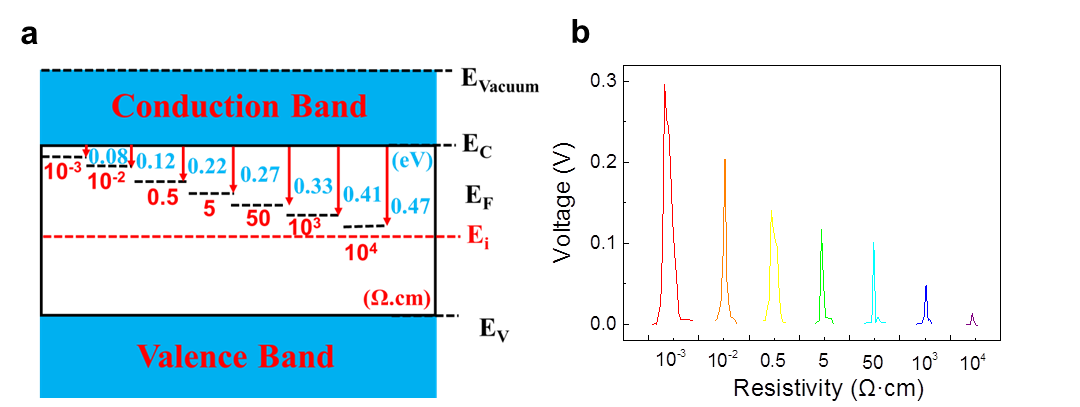


**Figure S5.** The detail effect of different N-type silicon resistivity on voltage output. (a) The one-dimensional band calibration of the Fermi level of N-Si substrates with the resistivity of 0.001, 0.01, 0.5, 5, 50, 1000 and 10000 Ω·cm respectively. (b) the relationship between the voltage output signal and N-Si substrate with different resistivity (keep the resistivity of P-type silicon unchanged at 0.001 Ω·cm).

Figure S6.


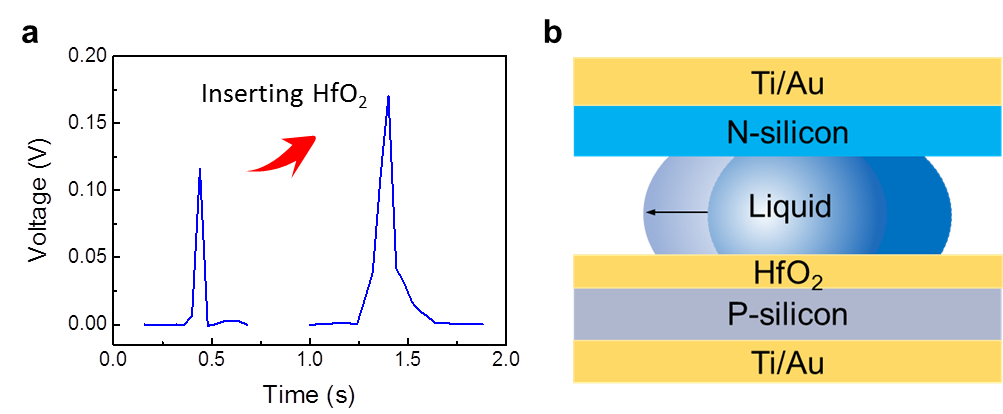


**Figure S6.** Optimization by increasing the interface barrier height. (a) Comparison of voltage output before and after adding hafnium oxide (HfO_2_) on the surface of N-type silicon (the volume of water is 50 μL, the sliding speed of the silicon wafer is 150 mm/s). (b) The structure diagram of the dynamic silicon-water-silicon generator after adding HfO_2_.

Figure S7.


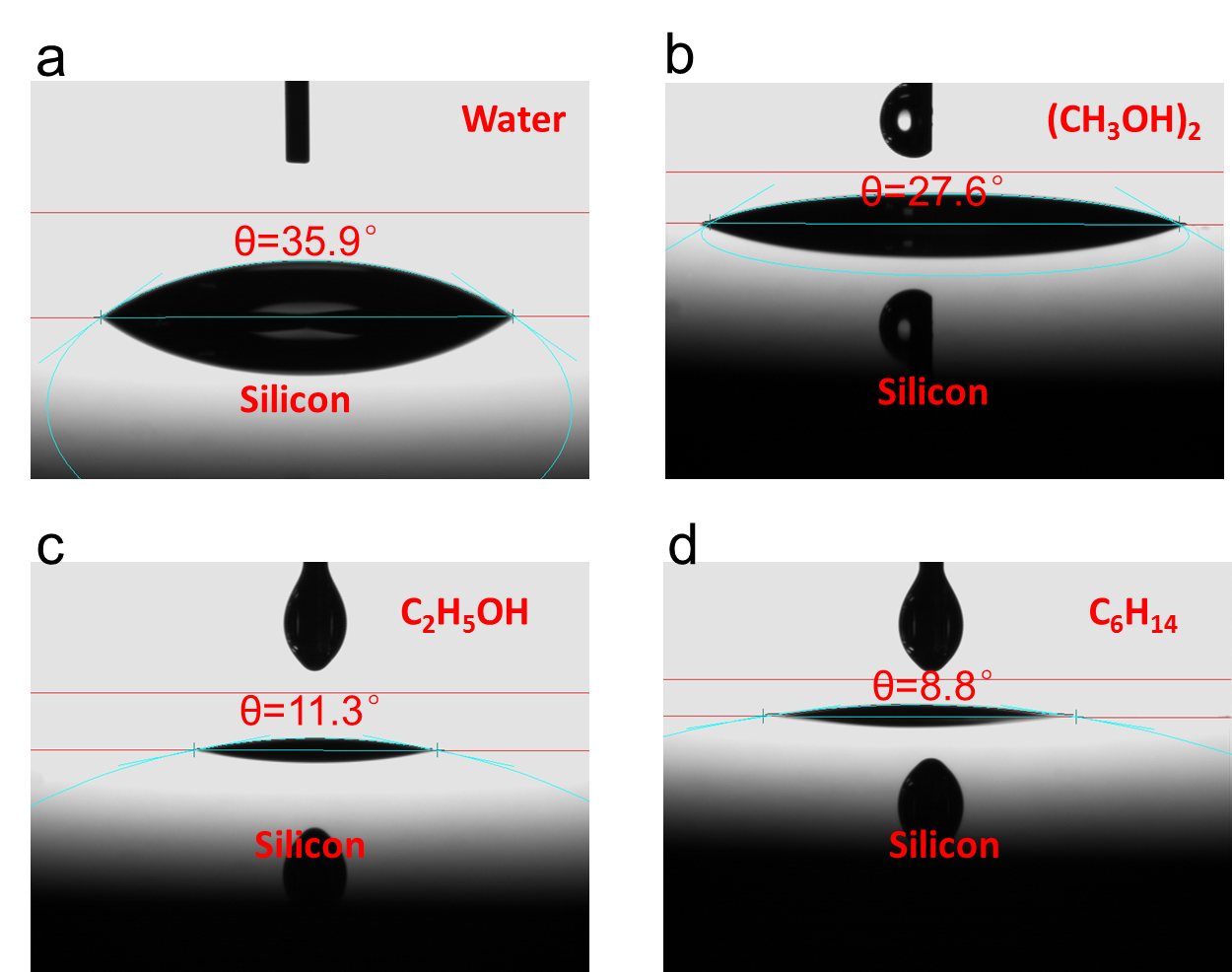


**Figure S7.** The contact angle of water, (CH_3_OH)_2_, C_2_H_5_OH and C_6_H_14_ on the Si substrate, indicating the surface wettability with different liquid.
